# Measuring the catastrophic cost of diagnosis, treatment, care, and support on people and families affected by tuberculosis in Iran and Afghanistan

**DOI:** 10.1016/j.ijregi.2025.100601

**Published:** 2025-02-18

**Authors:** Enayatollah Homaie Rad, Bilal Ahmad Rahimi, Minoo Alipouri-Sakha

**Affiliations:** 1 Social Determinants of Health Research Center, Trauma Institute, Guilan University of Medical Sciences, Rasht, Iran; 2 Faculty of Medicine, Kandahar University, Kandahar, Afghanistan; 3 Deputy of Health, Iran University of Medical Sciences, Tehran, Iran

**Keywords:** Tuberculosis, Catastrophic costs, Iran, Afghanistan, Direct costs, Indirect costs

## Abstract

**Objectives:**

A key goal of the World Health Organization's End Tuberculosis (TB) Plan was to eliminate TB-related catastrophic costs by 2030. This study aimed to measure the achievement of two countries (Iran and Afghanistan) in this plan.

**Design or Methods:**

Using a random cluster sampling, 649 patients with TB were interviewed in 2020. This study calculated the direct, indirect, and catastrophic costs for Iran and Afghanistan and applied regression estimators and a sensitivity analysis.

**Results:**

The results showed that 49% of households in Afghanistan and 20% of households in Iran faced TB-related catastrophic costs. Being in multidrug-resistant treatment (odds ratio [OR] = 3.670) was related to facing catastrophic costs. Households with a female (OR = 0.532), being an Iran resident (OR = 0.429), and being a university degree–holder (OR = 0.284) patient with TB had a lower likelihood of facing catastrophic costs.

**Conclusion:**

The findings of this study showed that these two countries are far from the goals of the World Health Organization for having zero TB-related catastrophic costs.

## Highlights

• Tuberculosis (TB)-related catastrophic costs in Iran and Afghanistan are 20% and 49%, respectively.
• The likelihood of facing catastrophic costs was higher in households with multidrug-resistant patients.
• Both countries are far from achieving a goal of the End TB Strategy, which was to eliminate the catastrophic costs of TB.

## Introduction

Tuberculosis (TB) remains one of the top 10 causes of death worldwide. In 2018, there were an estimated 10 million new TB cases worldwide [1]. The global average was about 130 TB cases per 100,000 people [2]. This means that progress is too slow to meet the goals of the End TB Strategy and the Sustainable Development Goals. They aim to end the TB epidemic by 2030 [2]. TB incidence is high in the eastern Mediterranean region (115 per 100,000 population) [3]. The association between poverty and TB is well-established. Poverty leads to TB, which, in turn, aggravates poverty [4].

The treatment for TB is very expensive. For example, the cost of treating a new smear-positive pulmonary TB patient was US $1409 in Iran in 2016 [5]. In the United States, the average direct cost of treating and managing a TB case was US $34,600 in 2015 [6]. The cost of TB is not imposed to the patient only, but all the family members are affected by the treatment costs. Indirect medical costs include absenteeism, discrimination, and stigma owing to lost earnings are some of them [7]. They also include the costs of health facility visits, hospital stays, and lost wages from being unable to work. Finally, they include the loss of home productivity for families because of the illness [[7], [8], [9], [10]]. Studies on costs related to TB showed that indirect costs were much higher than direct ones. A study in the Netherlands estimated indirect costs at €2603 and direct costs at €353 [10]. In a Chinese study, costs amounted to 4595.4 and 1180.1 Chinese Yuan, respectively [11].

TB-related cost burdens households, causing more poverty. Today, financial protection against health costs is vital. It prevents financial hardship for households. Health systems must protect those hit hard by out-of-pocket health costs. Health systems recognize the lack of financial protection as a health system failure. The catastrophic expenditure approach estimates the burden of health expenditure on households. The catastrophic expenditure measures how many households fall into poverty because of the disease costs. One goal of the End TB Strategy is to have 0% of TB-affected households face catastrophic costs by 2030 [3]. The World Bank and World Health Organization established a population-based measure of catastrophic expenditures. It is not comparable to the “TB catastrophic total costs” indicator of the End TB Strategy [12]. The TB catastrophic costs indicator includes direct medical and nonmedical costs and indirect costs, whereas the catastrophic expenditures are only direct out-of-pocket payments for health care, as the World Bank defines [13]. Indirect costs measure lost time and income from TB care-seeking and hospitalization. They do not capture the value of pain or illness [14]. Some studies have been carried out to estimate the prevalence of TB costs worldwide. However, few studies have analyzed it in the eastern Mediterranean and the progress toward the End TB Strategy by 2030 in the region. Therefore, this study calculated the catastrophic costs of TB in Iran and Afghanistan. The two countries are at opposite ends of the spectrum in TB burden in the eastern Mediterranean region. To the best of our knowledge, the TB-related catastrophic costs are not calculated in these two countries, and, for the first time, we tried the find new evidences about the goals of the End TB Strategy in these two countries.

## Methods

This study had three phases. First, the TB catastrophic costs’ questionnaire, recommended by the World Health Organization, was translated from English to local languages (Persian and Pashto). Two expert native speakers performed the translation. Then, three health experts and two patients with TB tested its content validity. Finally, the data were gathered.

This study applied a random cluster sampling. A total of 347 patients in Guilan, Iran and 302 in Kandahar, Afghanistan were randomly selected from the total patients with TB lists. For all patients with TB who agreed to join the study, an interview was scheduled. After confirmation of informed consent, the process of questioning was started. The process continued until the final sample was reached. The phone numbers or addresses of all the patients with TB from each country was gathered and they were called by phone for the questioning. If a case refused to participate in questioning or did not answer to the phone calls, it was removed from the study and a new case replaced it by random selection process. Trained interviewers were sent to the addresses of patients, and the interview was scheduled. The questionnaire used for data gathering contained the following:

• Pre-interview questions (patient info),
• Inclusion and exclusion criteria,
• Informed consent,
• A treatment phase checklist,
• An overview of TB treatments up to 2 years before the current treatment, and
• All costs before the current treatment.

The costs included health-seeking, diagnostic, and other costs. They also include costs before the current TB treatment. Patients were asked about their income before diagnosis of TB, any changes after, and their household's socio-economic status. They were also asked about their social position and health insurance. The informal sector is strong in Iran and Afghanistan. Therefore, the monthly self-reported household income may be biased. To avoid bias, the household annual income based on asset ownership was also used to calculate the monthly income. First, important asset information was asked in the questionnaire. Then, using data from two 2019 surveys, regression models were estimated. The first was the Iranian Statistical Center's household income and expenditures survey. The second was the Asia Foundation's survey of Afghan people. Using the estimated coefficients, the predicted income was calculated from the asset variables. The catastrophic costs faced by families were calculated using the following formula:

$$T=\frac{\sum OOPM+OOPN+IN}{y}$$

Where OOPM denotes direct nonmedical costs related to TB, OOPS denotes nonmedical costs related to TB, and IN denotes income loss related to TB. In addition, y is the household annual income before the TB contraction. If T exceeds 0.2, the family will face catastrophic TB costs. The frequency and prevalence of catastrophic costs were calculated for Iran and Afghanistan. A logistic regression model was used to find the relationship between variables and households facing catastrophic costs.

The informal sectors of two countries are not small. Many of the households do not like to disclose their real income. Therefore, using self-perceived income for calculating catastrophic costs might lead to bias. To avoid bias, the catastrophic costs were calculated using income from an indirect regression model. To do so, we used two income surveys in Iran and Afghanistan and predicted the income by adding the household living assets in the income predictor model. They were then compared with the catastrophic costs calculated using direct self-reported income. Furthermore, the results were estimated in different percentages of patients with multidrug-resistant (MDR) TB. The Iranian rial to US dollar exchange rate was 285,000 Iranian rials, and the afghanis to US dollar exchange rate was 81.50 afghanis at the time of study. We conducted the analysis using STATA SE v 14.1, Excel, and SPSS v 21 software. The deputy director of research at Guilan University of Medical Sciences confirmed the study's ethics (Ethics Code: IR.GUMS.REC.1400.272).

## Results

### *Descriptive statistics*

Table 1 shows descriptive statistics. As shown in the table, 29.8% and 29.11% of Afghan and Iranian patients were MDR, respectively. In Afghanistan, 51% of patients were illiterate. A total of 43% had a primary, 3% a secondary, 1% a high, and 2% an academic education. In Iran, 18% were illiterate. A total of 17.8% had a primary school degree. A total of 14.41% had a secondary school degree. A total of 31.9% had a high school degree. A total of 17.58% had an academic degree. Furthermore, 52.98% and 32.56% of Afghan and Iranian patients, respectively, were male. A total of 53.31% of Afghan patients said their living conditions did not change after TB. A total of 37.75% said they became poorer. A total of 8.94% said they became very poor. For Iranian patients, 64.5% said their living conditions did not change. A total of 15.8% became poor and 19.6% became very poor.

**Table 1:** Descriptive statistics.

Variable	Afghanistan		Iran		Total	
Multidrug-resistant	Frequency	Percentage	Frequency	Percentage	Frequency	Percentage
No	212	70.20	246	70.89	458	70.57
Yes	90	29.80	101	29.11	191	29.43
Total	302	100.00	347	100.00	649	100.00
**Education**						
Illiterate	153	51.00	63	18.16	216	33.38
Primary	129	43.00	62	17.87	191	29.52
Secondary	9	3.00	50	14.41	59	9.12
High school	3	1.00	111	31.99	114	17.62
University	6	2.00	61	17.58	67	10.36
Total	300	100.00	347	100.00	647	100.00
**Gender**						
Male	160	52.98	113	32.56	273	42.06
Female	142	47.02	234	67.44	376	57.94
Total	302	100.00	347	100.00	649	100.00
**Living condition after tuberculosis**						
Nothing changed	161	53.31	224	64.5	385	59.3
Poor	114	37.75	55	15.8	169	26
Very poor	27	8.94	68	19.6	95	14.6
Total	302	100.00	347	100.00	649	100.00
Mean age (SD)	35.711	1.263	48.184	0.8945	42.3805	0.80

### *Tuberculosis-related costs and catastrophic costs in Iran and Afghanistan*

Table 2 shows the results of direct, indirect, and household annual income in US dollars. As shown in the table, the direct and indirect costs of TB in Iran are higher than in Afghanistan, but the household annual income in Afghanistan is higher than in Iran.

**Table 2:** Direct, indirect, and household annual income in US dollars.

Variable	Mean	Standard error	95% lower limit	95% upper limit
Afghanistan				
Direct costs	185.1608	11.0947	163.3393	206.9824
Indirect costs	55.04016	6.834606	41.59756	68.48276
Household annual income	2618.146	71.72842	2477.067	2759.225
Iran				
Direct costs	389.2073	26.36602	337.3222	441.0923
Indirect costs	181.1719	8.76771	163.9181	198.4257
Household annual income	2056.944	39.62115	1978.975	2134.914

**Figure 1:**
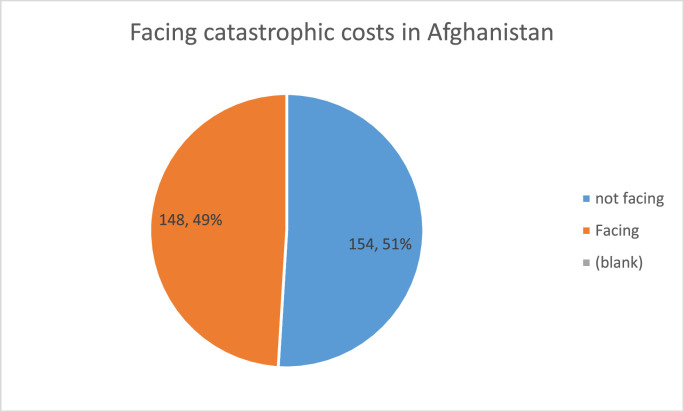
Percentage of catastrophic costs of tuberculosis in the Afghan population.

**Figure 2:**
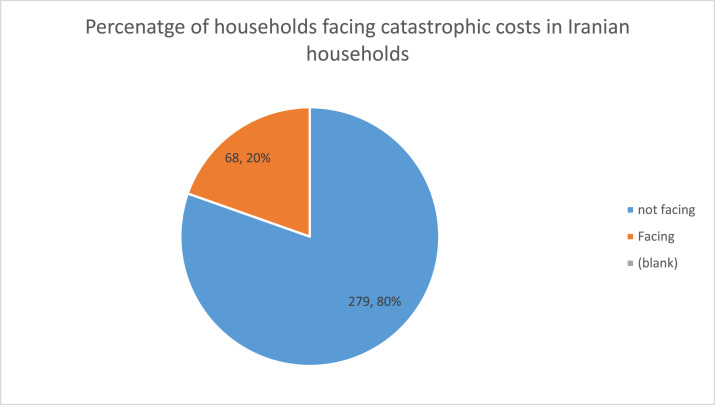
The results of facing tuberculosis-related catastrophic costs in Iranian households.

Figure 1 shows the percent of Afghans facing TB-related costs. It uses 20% of the annual income as the cutoff. Of the 302 households in the study, 148 (49%) faced catastrophic costs. The other 154 (51%) did not. Figure 2 shows the results of facing TB-related catastrophic costs in Iranian households. The figure shows that, of the 346 new households, 68 (19.6%) faced catastrophic costs from TB. The other 279 did not.

### *Regression analysis*

Table 3 shows the results of the regression estimations to find the relationship between different factors and facing catastrophic health costs. The dependent variable of the model is being faced catastrophic health expenditures or not. The table shows that households with patients with MDR TB faced higher catastrophic costs (odds ratio [OR] = 3.670, *P* <0.001). Education status was also related to catastrophic costs. The OR of the higher-educated groups is smaller than the lower-educated groups. This means they have a lower incidence of catastrophic costs. Furthermore, if the patient is female, the likelihood of facing catastrophic health costs in the family is lower (OR = 0.532, *P* = 0.001). Age was not significantly related to facing catastrophic costs. Patients who lived in Iran faced lower catastrophic health costs (OR = 0.429, *P* = 0.001).

**Table 3:** The results of the regression model show the relationship between different factors and facing catastrophic health costs.

Variable	Odds ratio	Standard error	*P*-value	95% lower limit	95% upper limit
Multidrug-resistant (yes)	3.670	0.748	0.000	2.461	5.473
Education					
Primary	0.599	0.132	0.020	0.389	0.922
Secondary	0.494	0.187	0.062	0.235	1.037
High school	0.336	0.123	0.003	0.164	0.690
University	0.284	0.117	0.002	0.126	0.637
Sex (female)	0.532	0.102	0.001	0.365	0.775
Age	0.993	0.005	0.170	0.984	1.003
Country (Iran)	0.429	0.108	0.001	0.262	0.701
Constant variable	1.486	0.408	0.149	0.867	2.546

### *Sensitivity analysis*

#### Using self-perceived household income instead of estimated income

For the sensitivity analysis, we added self-perceived income instead of estimated income for the data. Using self-reported household income, the catastrophic health cost rate was 31.4%. The results are presented in Table 4. As shown in the table, the two methods were agreed upon in 214 households about not facing catastrophic costs and 44 about facing catastrophic costs.

**Table 4:** Agreement of two methods for facing catastrophic costs because of tuberculosis.

	Self-perceived income			
		No	Yes	Total
Estimated income	No	214	65	279
	Yes	24	44	68
	Total	238	109	347

#### Changing in the percentage of multidrug-resistant patients

The regression model shows that MDR treatment greatly affects catastrophic expenditures. Suppose that x is the percentage of patients with MDR TB and y is the percentage of non-MDR ones. This phrase can be written as follows:

x+y=1

In addition, the total population's (k) catastrophic costs have two parts. MDR has 50.7% catastrophic costs, whereas non-MDR has 25.9%. Therefore, the percentage of catastrophic costs in the total population can be written as follows:

0.507x+0.259y=k

In addition, y can be rewritten as 1 − x, therefore:

0.507x+0.259(1−x)=k

0.507x−0.259x=k−0.259

0.248x=k−0.259

k=0.248x+0.2590<x<1

**Figure 3:**
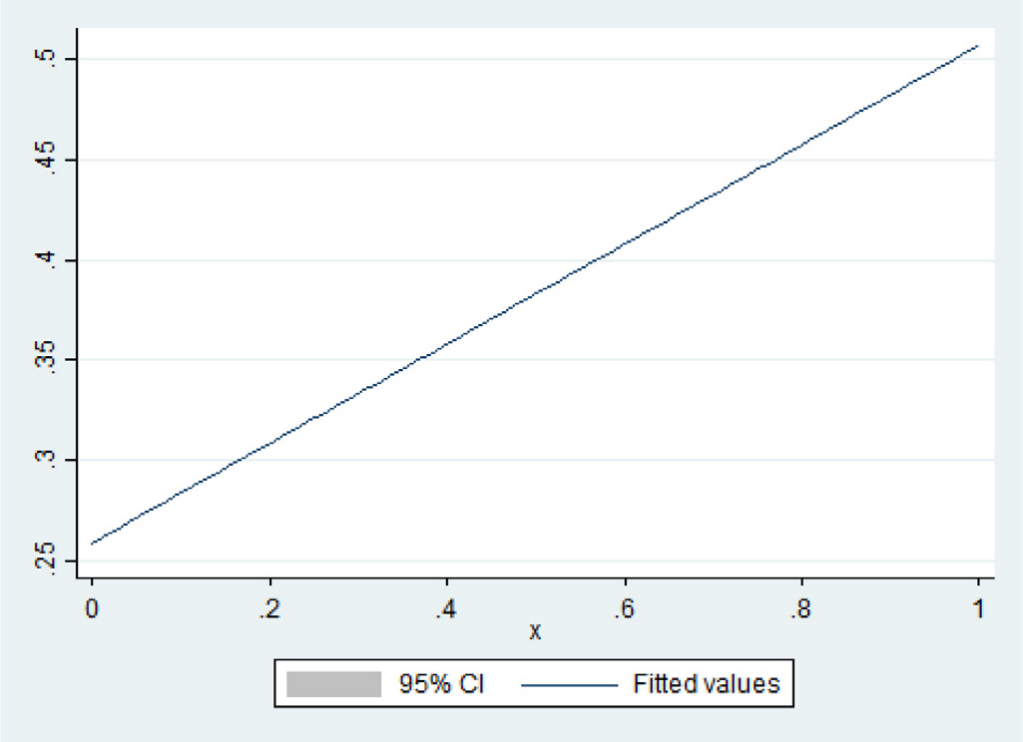
Simulation of changing in multidrug-resistant TB and facing catastrophic costs. CI, confidence interval.

Figure 3 shows the findings of the mentioned equation using 10,000 simulations. As shown in the figure, k has a range of 25-50%.

## Discussion

This study aimed to find the achievements of Iran and Afghanistan, in the eastern Mediterranean region, in reducing the costs of TB. Both these countries are facing international and national economic crises. Iran is under the sanctions of the United States and cannot export oil to other countries. Afghanistan and Iran were faced with a budget deficit in the fiscal year of 2021, which can affect health care provision indicators such as catastrophic costs [[15], [16], [17]]. TB control in Afghanistan is poor. It has one of the highest rates of the disease in the eastern Mediterranean and the world [[18], [19], [20]].

The elimination of catastrophic costs of TB is one of the main goals of the End TB Strategy [3] and it must be decreased to zero by 2030 [21]. Because of this strategy, many efforts have been made by governments to decrease out-of-pocket payments of TB treatment, improve health insurance coverage, and provide subsidies to the families facing TB, as well as achieving Universal Health Coverage [18]. However, this study found that Iran and Afghanistan are far from meeting the World Health Organization's goals.

A 2018 study in Nepal found that 53% of households faced catastrophic costs at a 20% threshold [22]. A study in Puducherry, India found that 7.8% and 16.7% of households faced catastrophic payments at 20% and 10% cut-off points, respectively [23]. A 2016 study in Vietnam found that 63% of households spent over 20% of their annual income on TB treatment and drugs [24]. A 2018 study in China found that catastrophic costs were 37.1% in all households and 69.6% in MDR households [25]. A study in Myanmar from 2015 to 2016 found that 60% of households faced catastrophic health costs when using 20% of their annual income as the cutoff [26].

The results of this study showed that patients with MDR TB are highly vulnerable to catastrophic health costs. The sensitivity analysis showed that an increase in the number of patients with MDR TB can lead to catastrophic costs. The governments must notice these patients and decrease the out-of-pocket cost of treatment. Treatment of patients with TB is not limited to free drugs and free physician consultations or hospital costs. Patients are burdened by many other costs, such as job loss costs, travel costs, nutrition costs, etc., which are not seen by the governments. The study found that Iranian patients with TB had higher treatment costs and lower incomes. However, they faced lower catastrophic costs from TB. It is highly related to the difference in income inequity between the two countries. Afghanistan's poor patient population was at a higher percentage than Iran's. Therefore, they faced more catastrophic costs from TB. The government can pay subsidies for the poor to improve their living conditions.

This study has some limitations. First, Afghans did not answer the income question in many cases. Therefore, we decided not to calculate the self-perceived catastrophic costs in Afghanistan. Second, we could not pool the results facing catastrophic costs in Iran and Afghanistan. The purchasing power is different in the two countries; thus, we decided to have a separate analysis. However, in the regression model, we pooled the data and added a dummy variable for the country. In future studies, we suggest to measure the time of identification of patients with TB and its relationship with TB surveillance and costs.

## Conclusion

The results of this study showed that Iran and Afghanistan, which are facing economic and political crises, are far from the End TB goals in catastrophic costs. To decrease the catastrophic costs of TB, the Ministries of Health in these two countries must focus on indirect costs of TB.

## Declarations of competing interest

The authors have no competing interests to declare.
